# Highly Rectifying Heterojunctions Formed by Annealed ZnO Nanorods on GaN Substrates

**DOI:** 10.3390/nano10030508

**Published:** 2020-03-11

**Authors:** Stanislav Tiagulskyi, Roman Yatskiv, Hana Faitová, Šárka Kučerová, David Roesel, Jan Vaniš, Jan Grym, Jozef Veselý

**Affiliations:** 1 Institute of Photonics and Electronics of the Czech Academy of Sciences, Chaberska 57, 18251 Prague 8, Czech Republic; yatskiv@ufe.cz (R.Y.); faitova@ufe.cz (H.F.); kucerova@ufe.cz (Š.K.); roesel@gmail.com (D.R.); vanis@ufe.cz (J.V.); 2 Faculty of Mathematics and Physics, Charles University, Ke Karlovu 3, 12116 Prague 2, Czech Republic; jozef.vesely@mff.cuni.cz

**Keywords:** ZnO nanorods, nanoscale heterojunctions, current-voltage characteristics, chemical bath deposition, annealing, focused ion beam patterning, nanoprobe in the scanning electron microscope

## Abstract

We study the effect of thermal annealing on the electrical properties of the nanoscale *p*-*n* heterojunctions based on single *n*-type ZnO nanorods on *p*-type GaN substrates. The ZnO nanorods are prepared by chemical bath deposition on both plain GaN substrates and on the substrates locally patterned by focused ion beam lithography. Electrical properties of single nanorod heterojunctions are measured with a nanoprobe in the vacuum chamber of a scanning electron microscope. The focused ion beam lithography provides a uniform nucleation of ZnO, which results in a uniform growth of ZnO nanorods. The specific configuration of the interface between the ZnO nanorods and GaN substrate created by the focused ion beam suppresses the surface leakage current and improves the current-voltage characteristics. Further improvement of the electrical characteristics is achieved by annealing of the structures in nitrogen, which limits the defect-mediated leakage current and increases the carrier injection efficiency.

## 1. Introduction

ZnO is rich in applications over a wide range of industrial areas. For modern optoelectronics ZnO offers several advantages over other wide-bandgap materials such as the low cost and wide availability, environmental safety, simple crystal-growth technologies, high thermal stability, high exciton binding energy (60 meV), and direct band gap (3.37 eV) [1]. The high exciton binding energy and wide direct band gap are particularly important to pave the way for manufacturing of blue/ultraviolet light emitting devices (LEDs) [2]. Reproducible *p*-type doping is beyond the reach in ZnO [3], which limits the use of ZnO in *p*-*n* homojunction devices. Therefore, light-emitting heterostructures are often designed on the basis of *n*-type ZnO with other *p*-type materials [4,5,6]. Among them, thin film *n*-ZnO/*p*-GaN heterojunctions belong to the most investigated systems, because of the same wurtzite hexagonal lattices and almost equal band gaps of ZnO (3.37 eV) and GaN (3.39 eV). The devices based on thin film heterostructures, however, suffer from the evolution of the strain at the interface and from the existence of the band offset, which reduces the efficiency of carrier injection [7].

To overcome the limitations of thin film technology, the heterostructures can be realized in the form of ZnO nanorods or nanowires on GaN substrates [8,9,10]. The nanoscaled interface allows for elastic relaxation of the strain and for more efficient carrier injection and light extraction [11]. Park et al. [12] reported a strong electroluminescence from the arrays of vertically aligned nanorods grown by metalorganic chemical vapour deposition (MOCVD). The efficiency of the carrier injection was enhanced in nanosized junctions between the *n*-type ZnO nanorod and the *p*-type GaN substrate. Shi et al. reported on ZnO nanowire-based LEDs prepared by MOCVD on *p*-type GaN substrates with efficient electroluminescence and rectifying behavior with the turn-on voltage of 3.7 V [13]. The electroluminescence was attributed to near-band-edge emission in ZnO and acceptor-to-band transition in GaN depending on the region of charge carrier recombination. Zhu et al. [14] presented optical properties of ZnO microrods grown by vapor transport and reported on whispering gallery mode lasing. The forward biased ZnO microrod/GaN UV LEDs demonstrated good performance (*V_on_* ~ 2.6 V, rectification ratio ~106) and bright electroluminescence with the spectra that consisted of three distinct electron-hole recombination processes—near band edge emission in ZnO, interface recombination, and band-to-acceptor transitions in GaN substrate. The arrays of ZnO nanorods were shown to form high quality channels for electron injection and light extraction in nanorod-based LEDs. The next state-of-art structure was the highly ordered arrays of nanorods on pre-patterned substrates. The combination of the optical or electron beam lithography and solution growth of ZnO [15] were employed to achieve better control over the nanorod synthesis [16]. Good reproducibility of every single nano-heterojunction in the array led to homogeneous light emission and better control over charge transport in the whole array of the nanorods [10,17]. Moreover, the well-ordered periodic arrays of the light guides can form two-dimensional photonic crystal with controlled out-coupling of photons [18].

Solution growth of ZnO nanorods takes place at low temperature, which gives the rise of native defect formation in the ZnO lattice because of their low formation energy [2]. The lattice of solution grown ZnO is usually nonstoichiometric with the excess of Zn. Point and structural defects in ZnO lattice strongly influence morphological, optical, and electrical properties of as-grown ZnO [19]. The properties of LEDs based on the nanorod arrays are strongly related to the quality of the *p*-*n* heterojunction interface. In as-grown diodes, the large leakage current was attributed to defect mediated tunneling via the defects and trap centers near the *p*-*n* heterojunction interface [20]. The defects and the corresponding interface states were frequently reported to be responsible for the degradation of the light emission efficiency in the nanorod-based LEDs [13,18,21,22]. Post-annealing treatment is an effective method to enhance the crystalline quality of the nanorods and to reduce the density of interfacial defects with the potential to greatly enhance the diode performance of the structures. Previous results showed that annealing in nitrogen ambient enables to modify the deep level defects, to enhance the UV light emission, and to improve the diode performance of ZnO-nanorod based heterostructures [19,23,24,25,26,27].

The diodes based on ZnO nanorod/GaN interfaces often suffer from insufficient charge separation. Electrons directly tunnel through the ZnO/GaN interface, which leads to symmetrical nonlinear *I*–*V* characteristics with a high reverse bias current and bias-dependent near-band-edge emission form the GaN substrate rather than from the ZnO nanorods [17,22,28]. To solve this issue more complex structures with additional oxide layers were reported to achieve proper performance of the nanorod-based LEDs [28,29,30].

Further downscaling of optoelectronic devices requires deep understanding of electrical properties of nanoscale heterojunctions based on a single nanorod or nanowire. Despite the fact that LEDs based on the ZnO nanorod arrays have been extensively studied, the current-voltage (*I*–*V*) characteristics of single-nanorod heterojunctions were reported only recently by our research group [31]. The techniques of the investigation of electrical characteristics of individual nanostructures using a nanoprobe in atomic force microscope [32], scanning transmission electron microscope [33], or scanning electron microscope [34,35,36] have recently received particular attention. Vertical geometry of the nanorods is compatible with the working conditions of optoelectronic devices (nano-LEDs, nanorod based solar cells, etc.), which further emphasizes the importance of the nanoprobe techniques. Moreover, the nanoprobe approach to contact individual nanostructures is less time-consuming in comparison with other techniques, such as electron beam lithography, where every single nanorod needs to be transferred to another substrate and only then the contacts are deposited by lithographic procedures [37,38].

In this paper, well-ordered *n*-ZnO nanorods were grown by chemical bath deposition on *p*-GaN substrates patterned using focused ion beam (FIB) lithography. The current-voltage characteristics of single nanorod heterojunctions were measured using the nanoprobe in the high vacuum chamber of a scanning electron microscope. Highly rectifying *I*–*V* characteristics are ascribed to the formation of nanoscale heterojunctions created between a single ZnO nanorod and the FIB-patterned GaN substrate. The performance of the heterojunctions is further improved by thermal annealing, which suppresses defect-assisted current leakage and increases the concentration of free carriers.

## 2. Materials and Methods 

The arrays of ZnO nanorods were grown by chemical bath deposition on *p*-GaN: Mg (HVPE; Kyma, Inc., Raleigh, NC, USA) epitaxial templates with the acceptor concentration *N_A_* ≈ 5 × 10^17^ cm^−3^ [15]. The GaN substrate was thoroughly cleaned with acetone in an ultrasonic bath with subsequent etching in concentrated (28%) ammonium hydroxide solution at 50 °C for 20 min. During the next step, a 200 nm thick layer of poly(methyl methacrylate) film (PMMA) was deposited on the substrate by spin-coating. An array of circular holes in the PMMA layer was fabricated using focused Ga^+^ ion beam (FIB) with the acceleration voltage 30 keV and the probe current 57 pA. The diameter of the hole at the interface with the GaN epitaxial layer was ~250 nm while the depth of the holes was ~300 nm.

**Figure 1:**
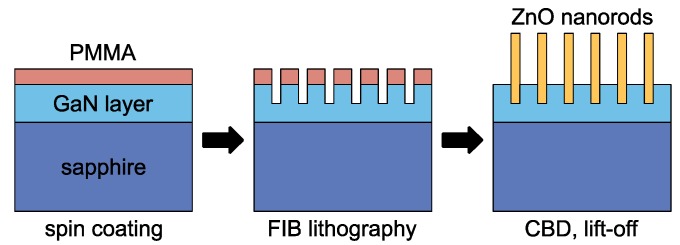
Flowchart of the fabrication process. The GaN epitaxial layer grown on sapphire substrate was covered by poly(methyl methacrylate) (PMMA) layer. First, the array of circular holes was created into the substrate by focused ion beam (FIB) lithography. Then, the ZnO nanorods were grown from the openings using chemical bath deposition (CBD) technique. Finally, the PMMA mask was removed.

The ZnO nanorods were grown from a 5 mM aqueous solution of zinc nitrate hexahydrate (which supplies the reaction with Zn ions) and hexamethylenetetramine (which supplies the reaction with OH^−^ ions). To activate the reaction, the temperature of the solution was increased to 95 °C for 2 h [39]. A standard lift-off procedure to remove the resist was performed after the growth process. The ZnO nanorods were also grown on plain (non-patterned) GaN substrates at identical growth conditions to clarify how the ion beam lithography changes the properties of the nanoscaled heterojunctions. A simplified flowchart of the fabrication process is shown in Figure 1. The as-grown nanorod arrays were further annealed at 200 °C, 400 °C, and 600 °C for 1 h in nitrogen atmosphere to enhance the crystalline quality and electrical properties of the structures.

**Figure 2:**
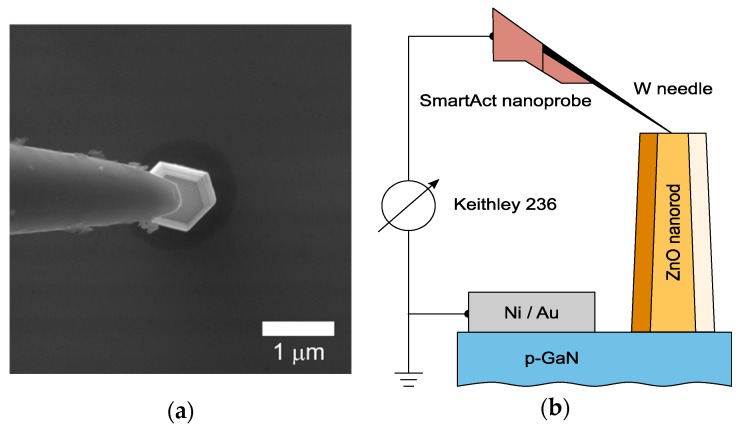
(**a**) SEM image of the nanomanipulator needle in contact with a single ZnO nanorod. (**b**) The experimental set-up for the electrical probing of a single nanorod in SEM.

The morphology of the ZnO nanorods was observed by scanning electron microscopy (SEM) (Lyra 3 GM FIB/SEM, TESCAN, Brno, Czech Republic) while the interface between the GaN substrate and the ZnO nanorods was studied by transmission electron microscopy (TEM) (2200FS HRTEM, JEOL Ltd., Tokyo, Japan). The *I*–*V* characteristics of the nanoscale heterojunctions formed by a single ZnO nanorod on GaN substrate were measured in the SEM chamber (Figure 2). The samples were stored in the SEM chamber for several hours before the measurements to reduce the impact of persistent photoconductivity on the electrical properties of ZnO nanorod/GaN heterostructures [29]. A tungsten needle of a SmarAct nanoprobe served as a top ohmic contact. The tip of the needle was first cleaned using the focused ion beam to remove the native tungsten oxide to provide reproducible *I–V* measurements. The bottom ohmic contact was deposited on the cleaned GaN substrate by vacuum evaporation of a 50 nm layer of Ni covered with a 40 nm layer of Au with subsequent annealing at 500 °C for 1 h in nitrogen. The *I*–*V* characteristics were measured by applying a linear voltage sweep to the top needle contact while the bottom contact was grounded. Within this paper when a negative voltage is applied to the top contact, the *p*-*n* junction between the *n*-ZnO nanorod and *p*-GaN substrate is further considered as forward biased and vice versa.

## 3. Results

### 3.1. Growth of ZnO Nanorods on FIB-Patterned Substrates

**Figure 3:**
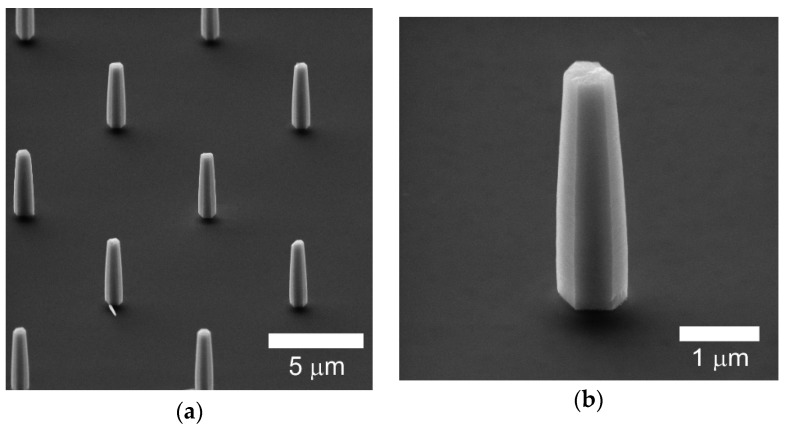
(**a**) SEM image of the array of ZnO nanorods and (**b**) of a single ZnO nanorod on the GaN substrate patterned by FIB.

Figure 3 shows SEM images of the arrays of ZnO nanorods of a regular hexagonal shape grown by chemical bath deposition on the FIB-patterned GaN substrates. Each nanorod in the array has identical dimensions with the length *L* ≈ 4 µm and radius *r* ≈ 300 nm. For comparison, the nanorods were also grown on plain GaN substrates under the same growth conditions; however, with significant size dispersion of the length *L* ≈ 2 – 10 µm and diameter *r* ≈ 100 nm – 2 µm (not shown).

### 3.2. Electrical Properties

**Figure 4:**
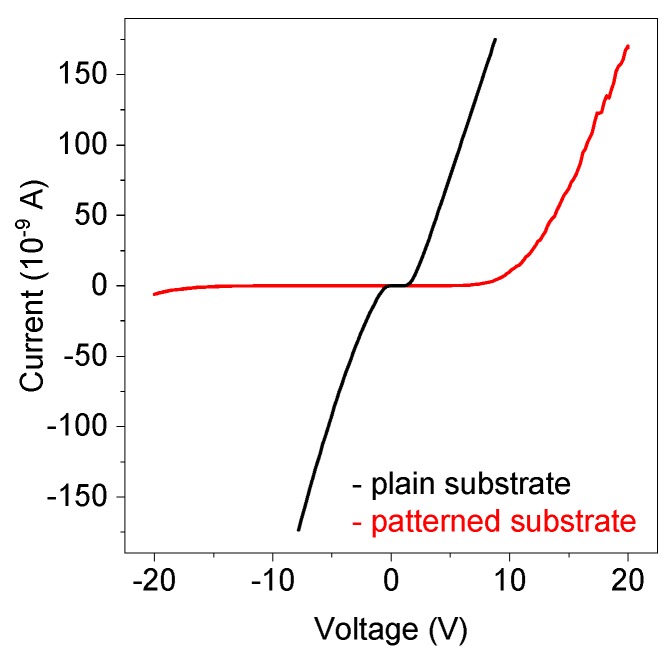
*I*–*V* characteristics of single nanorod *n*-ZnO/*p*-GaN heterojunctions that were fabricated on both the plain and FIB-patterned substrates. The patterned structure exhibits a rectifying diode-like *I*–*V* characteristic. The plain structures show a nonlinear almost symmetrical *I*–*V* characteristic.

Figure 4 shows the *I*–*V* characteristics of a single ZnO nanorod on the plain GaN substrate and on the GaN substrate patterned by FIB. The *I*–*V* characteristics of the plain structure is nonlinear almost symmetrical with a large leakage current under the reverse bias. On the contrary, the *I*–*V* characteristic of the FIB-patterned structure is highly rectifying with the forward cut-in voltage *V_on_* ~ 3 V, the reverse breakdown voltage *V_br_* ~ −4.6 V, and the rectification ratio of 68 at ± 4 V.

**Figure 5:**
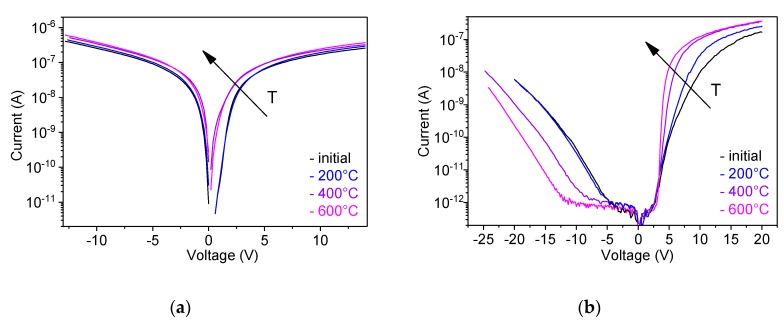
Semi-logarithmic *I*–*V* plots at different annealing temperatures for single nanorod *n*-ZnO/*p*-GaN heterojunctions, (**a**) gown on the plain substrate and (**b**) FIB-patterned substrate. A significant improvement of the diode performance is observed on the FIB-patterned substrates when the annealing temperature is 400 °C or higher.

Figure 5 shows semi-logarithmic *I*–*V* characteristics of the plain and FIB-patterned structures after annealing at 200 °C, 400 °C, and 600 °C for 1 h in nitrogen gas atmosphere. The annealing has a clearly observable impact on the *I–V* characteristics when the temperature is 400 °C or higher and is more efficient for the FIB-patterned structures. For the plain structures the current increased under both polarities of the applied voltage and the forward bias *I*–*V* characteristics became less linear, as the annealing temperature was increased (Figure 5a). A significant improvement of the diode performance was observed for the annealed FIB-patterned structures. With increasing annealing temperature both the slope of the forward bias *I–V* characteristics and the reverse breakdown voltage significantly increased and the rectification ratio reached 1700 (Figure 5b). To the extent of our knowledge, these structures have the best diode performance ever reported for single ZnO nanorod heterojunctions.

## 4. Discussion

### 4.1. The Impact of FIB on the Nucleation of ZnO Nanorods and on the Electrical Properties of the ZnO/GaN Heterojunctions

**Figure 6:**
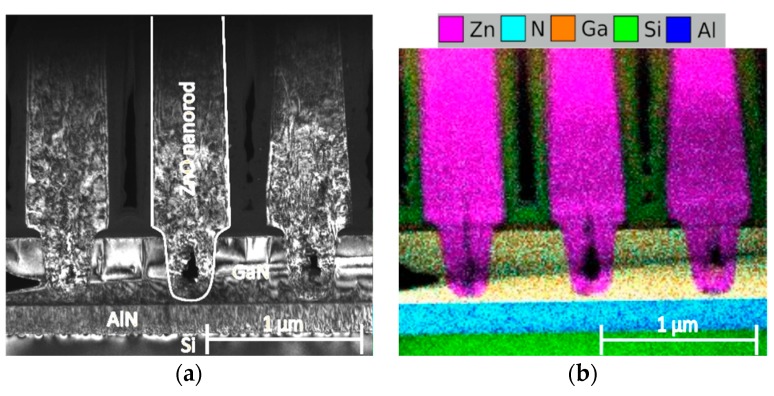
(**a**) TEM image of the interface between the ZnO nanorod and GaN substrate; (**b**) Energy-dispersive X-ray Spectroscopy analysis (EDX) of the interface between the ZnO nanorods and the GaN substrate.

The surface non-uniformity of the GaN epitaxial layers does not enable to use more conventional electron beam lithography to pattern the substrate, because of the non-uniform nucleation of ZnO, which leads to a broad distribution of sizes of the nanorods. The FIB lithography creates trenches in the GaN substrate. Preliminary SEM and TEM analyses showed that the first crystallites of ZnO appear around the interface of GaN with PMMA, which further develop and merge into a nanotube with a facetted outer and rough inner surface. By lateral growth, the nanotube transforms into a nanorod often with a hollow left in the trench (Figure 6). The uniform nucleation is behind the excellent uniformity of the nanorods in the array as well as behind the improved electrical characteristics of the single nanorod heterojunctions. The interaction of FIB with the GaN surface is a complex process which requires a separate in-depth study. The nucleation of ZnO from the trenches milled by FIB is affected by dissociation of the surface nitrogen atoms on the GaN surface [8], amorphization of the GaN surface [40], implantation of Ga [41], and the localization of the nucleation to the interface of GaN with the resist [42].

**Figure 7:**
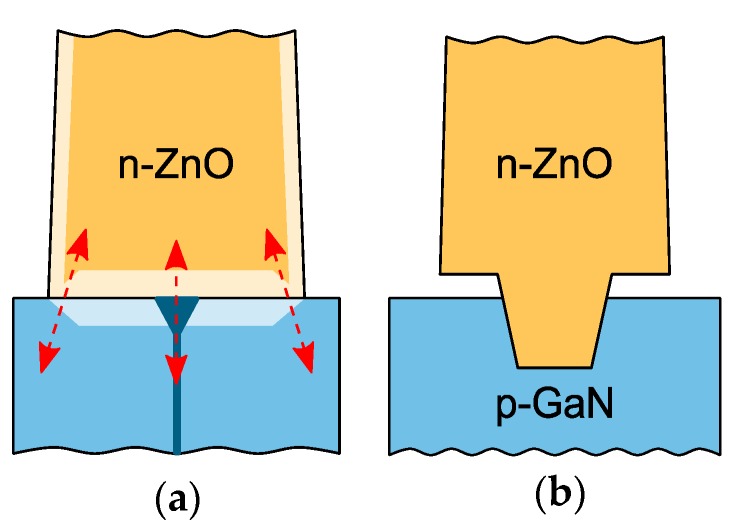
Schematic of the ZnO/GaN interface for the plain and patterned structures. (**a**) The two possible leakage paths through the interface of the plain substrate are depicted—the tunneling through a thinner barrier along the edge of the interface and the leakage caused by the inhomogeneity of the interface and by the extended structural defects of the GaN substrate. (**b**) On the FIB-patterned substrate the leakage current is suppressed, since the ZnO/GaN interface is formed only within the milled trench while the bottom edge of the ZnO nanorod is isolated from the GaN substrate due to lateral overgrowth of ZnO over the growth mask.

The configurations of both plain and patterned interfaces should be carefully considered to answer the question why the rectifying *I*–*V* characteristics are observed only on the patterned substrates. On the plain substrates, an axial ZnO/GaN heterointerface is formed (Figure 7a). The nucleation of ZnO crystallites starts randomly at several sites on the GaN substrate, which leads to nonhomogeneous ZnO/GaN interface. Symmetrical *I*–*V* characteristics of the plain structures are related to the large reverse bias leakage current caused by the tunneling through a thin barrier along the perimeter of axial interface [17,33]. Moreover, dislocations into GaN layer were reported as the nucleation sites for the growth of ZnO on GaN substrates [43]. These dislocations can strongly enhance the leakage current in GaN [44]. For the FIB-patterned substrate, the radial ZnO/GaN heterointerface is localized within the trench in the GaN substrate. The bottom edge of the ZnO nanorod is isolated from the GaN substrate because of the lateral overgrowth of ZnO over the PMMA mask and consequently the surface leakage is suppressed (Figure 7b).

**Figure 8:**
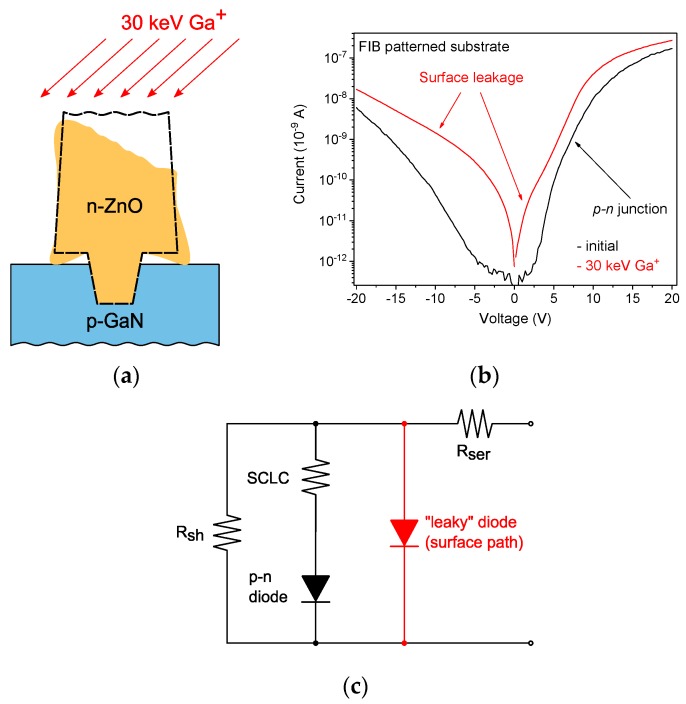
(**a**) Schematic of the cross-section of the of ZnO/GaN interface on FIB-patterned GaN substrates GaN with a restored conductive path after post-growth FIB milling. (**b**) Semi-logarithmic *I*–*V* plots for the single nanorod ZnO/GaN heterojunction on FIB-patterned GaN substrates before and after restoring the surface conductive path. (**c**) The electrical circuit of a single nanorod heterojunction with the additional surface leakage path.

Another confirmation of the key impact of the surface leakage on the *I*–*V* characteristics was obtained by FIB milling of the apex of the ZnO nanorods, which was originally performed with the aim to improve the top ohmic contact to ZnO. The surface conductive path was unintentionally restored by redeposition of ZnO sputtered by FIB (Figure 8a). As a result, the *I*–*V* characteristics became more symmetrical with both a large reverse bias leakage current and a decreased slope of the linear part of the semi-logarithmic forward *I*–*V* characteristic (Figure 8b). This behavior was attributed to a leaky diode in series with the *p*-*n* junction (Figure 8c) [45].

In summary, the FIB lithography provides a uniform nucleation of ZnO on the initially non-uniform GaN substrates without the need to deposit seed layers at the ZnO/GaN interface. The uniform nucleation results in the growth of highly uniform arrays of ZnO nanorods and the specific configuration of the interface strongly suppresses the surface leakage and thus improves the *I*–*V* characteristics.

### 4.2. The Effect of Thermal Annealing on the Electrical Properties of ZnO/GaN Nanoheterojunctions

Despite the fact that the *I*–*V* characteristics were significantly improved by FIB patterning of the substrate, the issue of a high concentration of defects in solution grown ZnO nanorods remained. To suppress defect-states-mediated leakage through the *p*-*n* junction interface, thermal annealing was performed in nitrogen at different temperatures. The annealing at 200 °C had only minor influence on the *I*–*V* characteristics, which is in accordance with previous reports that suggested temperatures higher than 300 °C to anneal defects in ZnO and to desorb surface species [46]. After annealing at 400 °C and 600 °C, the conductivity of the plain structures was moderately increased (Figure 5a). The moderate increase is related to increased concentration of free carriers caused by desorption of surface species [38]. However, narrowing of the surface depletion region enhances the surface leakage current along the edge of the interface. The deviation of the semi-logarithmic forward *I*–*V* characteristic from linearity and the decrease of the slope is a result of the additional leakage path that bypasses the *p*-*n* heterojunction. On FIB-patterned structures, the annealing strongly suppresses the defect-mediated leakage, which is confirmed by both increased slope of the semi-logarithmic forward *I*–*V* characteristic and increased the absolute value of the breakdown voltage at the reverse bias (|*V_br_*| = 12 V after annealing at 600 °C) (Figure 5b).

**Figure 9:**
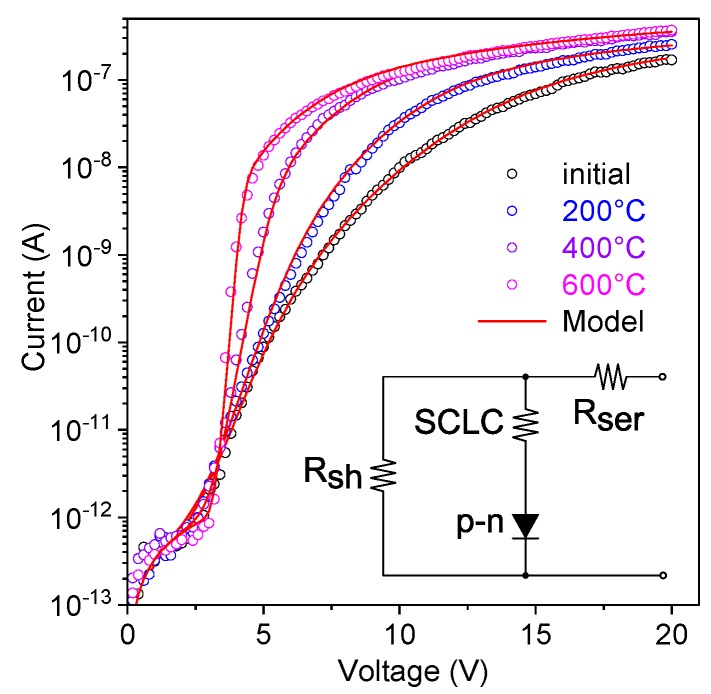
Experimental (symbols) and theoretically fitted (solid line) forward bias *I*–*V* curves for the annealed single nanorod ZnO/GaN heterojunction on the FIB-patterned substrate. The inset shows the equivalent electrical circuit of the single nanorod heterojunction used for the fitting.

To analyze the impact of annealing on the single nanorod-based heterojunction, theoretical modeling of the forward *I*–*V* characteristic was performed (Figure 9). The proposed model takes into account: (i) charge transport through the *p*-*n* heterojunction interface (the diode element in the equivalent circuit); (ii) the current flowing through the space charge region of the ZnO nanorod (SCLC resistor the equivalent circuit); (iii) the leakage current that bypasses the junction region; (iv) the resistance of the contacts and neutral regions of semiconductors (*R_ser_* resistor the equivalent circuit).

The current flowing through the *p*-*n* heterojunction is given by

(1) $$I_{p-n}=I_{0}\left(e^{AV}-1\right)$$

where *I_0_* is the reverse bias saturation current of the junction and A is the exponent coefficient. In the frame of the thermionic emission model the ideality factor of the *p*-*n* junction can be calculated by *η* = *q/AkT*, where *q* is the electron charge, *k* is the Boltzmann’s constant, and *T* is the temperature [45].

At a higher bias, when the *p*-*n* junction has negligible resistance, the resistance of the nanorod limits the charge transport. However, the *I*–*V* characteristics remain nonlinear at high bias, which suggests that the current is limited by the conductance of the space charge region. In the general case, the space charge limited current (SCLC) is given by:

(2) $$I_{SCLC}=kV^{\beta }$$

where *k* is the coefficient related to the length and conductivity of the current path and *β* is the power factor, which should be ~2 for trap-free space charge limited current, or >2 for a high density of deep traps [47].

The leakage current that bypasses the heterojunction follows the Ohm’s law:

(3) $$I_{shunt}=V/R_{sh}$$

where *R_sh_* is the resistivity of the shunting conductive path. 

According to Kirchhoff’s current law the elements described by Equations (1)–(3) connected in series with the resistance *R_ser_* give rise to the total current injected to the single nanorod ZnO/GaN heterojunctions:

(4) $$I=\frac{I_{0}\left(e^{A\left(V-IR_{ser}\right)}-1\right)k{\left(V-IR_{ser}\right)}^{\beta }}{I_{0}\left(e^{A\left(V-IR_{ser}\right)}-1\right)+k{\left(V-IR_{ser}\right)}^{\beta }}+\frac{\left(V-IR_{ser}\right)}{R_{sh}}$$

Figure 9 shows *I*–*V* characteristics theoretically modeled with equation (4) using *I_0_*, *A*, *k*, *β*, *R_ser_ R_sh_* as fitting variables. The modelled curves fit well the experimental *I*–*V* characteristics of single nanorod *n*-ZnO/*p*-GaN heterojunctions fabricated on FIB-patterned substrates after thermal annealing. The fitting variables and the ideality factor *η* (extracted from the coefficient *A*) are given in Table 1. The ideality factor *η* and the saturation current *I_0_* were significantly lowered after the high temperature annealing, which supports our claim that the defect-assisted tunneling was strongly suppressed. Despite the fact that the ideality factor has the lowest value ever reported for nanorod-based ZnO/GaN heterojunctions (*η* ≈ 4.5 after annealing at 600 °C), it remains too large for classical thermionic field emission model [45]. Therefore, typically for wide bandgap semiconductors, the tunneling-recombination process is considered as the main transport mechanism for the annealed *p*-*n* heterojunctions [48].

According to the model of the SCLC developed for the nanowires with the large aspect ratio (L/r ≥ 5) the Equation (2) can be rewritten as:

(5) $$I_{SCLC}=\theta \cdot {\left(r/L\right)}^{-2}\cdot \varepsilon \mu V^{2}/L^{3}$$

where *ε* is the dielectric constant, *μ* is the electron mobility, *V* is the applied bias voltage, *L* is the length of the nanorod, *r* is the radius of the nanorod, *θ* denotes the ratio of free to trapped charge, and ${\left(r/L\right)}^{-2}$ is the geometrical scaling factor related to the specific geometry of the nanorods [34,36].

Due to the presence of charge traps that are distributed over the band gap of the semiconductor the scaling exponent of the power-law *I*–*V* dependence increases. The power law region of the *I*–*V* characteristics with the scaling exponent larger than two is ascribed to the trap-filling limited (TFL) conduction mechanism. The θ coefficient from Equation (5) can be expressed as:

(6) $$\theta =\theta_{0}{\left(r/L\right)}^{-2}{\left(V/V_{c}\right)}^{\gamma }$$

where *θ*_0_ represents the fraction of free charge at the crossover to SCL range bias [35].

Combining Equations (5) and (6), we obtain the expression for the TFL conduction with the exponential distribution of traps:

(7) $$I_{TFL}=\theta_{0}{\left(V/V_{c}\right)}^{\gamma }\cdot {\left(r/L\right)}^{-2}\cdot \varepsilon \mu V^{2}/L^{3}$$

where *V*_c_ is the crossover voltage corresponding to the onset of SCL conduction, *θ*_0_ represents the fraction of free charge at the crossover voltage *V*_c_, the exponent *γ* is related to the characteristic temperature of the trap distribution $T_{C}$ as [47]:

(8) $$\gamma =T_{C}/T-1$$

Now we can consider the physical meaning of the fitting variables *k* and *β* from equation (4) taking into account the SCLC and TFL theories (Equations (5) and (7)). First, the increase of the pre-exponential coefficient *k* of the SCLC law denotes a larger ratio of free to trapped charge in the annealed structures. Loosely adsorbed surface species are desorbed after annealing and bound electrons are released. The released free electrons increase the free carrier concentration [20,38]. Second, regarding equation (7) we can express the fitting parameter *β* = *γ* + 2 and estimate the mean characteristic energy of charge traps below the conduction band edge *E_c_* − *E* as [47]:

(9) $$E_{c}-E=kT_{c}=kT\left(\gamma +1\right)$$

At 300 K the characteristic energy *kT*_c_ decreases from 120 meV for the as-grown samples to ≈ 100 meV for the samples annealed at 600 °C. For the ZnO layers annealed in nitrogen atmosphere, the traps located at such energy levels are usually attributed to Zn-sublattice defects, zinc interstitials (*I*_Zn_), and nitrogen on the substitutional oxygen site (*N*_o_) [26,49]. Moreover, Look et al. [50] proposed that the *I*_Zn_*–N*_O_ complex is the main shallow donor in ZnO annealed in nitrogen atmosphere. Therefore, it is reasonable to suggest that the increase of the free carrier concentration in the annealed samples is related to zinc interstitials (*I*_Zn_) or shallow donors stabilized in the form of the complex defects with nitrogen on the substitutional oxygen site (*N*_o_).

In summary, the annealing significantly improves the electrical performance of the nanorod-based *n*-ZnO/*p*-GaN heterojunctions. The defect-mediated leakage current is strongly suppressed and the carrier injection efficiency is enhanced by the increase of the carrier concertation in the conduction band related to both the desorption of surface acceptors and the formation of shallow donors in the annealed samples.

## 5. Conclusions

We investigated the impact of thermal annealing on the electrical properties of nanoscale *p*-*n* heterojunctions formed by a single *n*-type ZnO nanorod on *p*-type GaN substrates. The ZnO nanorods were grown by chemical bath deposition on conventional non-patterned GaN substrates and on the substrates locally patterned by focused ion beam lithography. The substrate modification by focused ion beam was shown to be crucial to achieve uniform nucleation and growth of ZnO nanorods on GaN and to form rectifying heterojunctions. The thermal annealing in nitrogen atmosphere significantly enhanced the diode performance by improving structural properties of the interface, which resulted in a strong decrease of the leakage current and in an enhanced efficiency of electron injection in the nanoscale heterojunctions. The equivalent electrical circuit was proposed to fit the measured current-voltage characteristics, from which the parameters characterizing charge transport through the heterojunction were extracted. The key parameters, such as the rectification ratio, the ideality factor, and the reverse bias breakdown voltage were shown to be largely improved after annealing.

## Figures and Tables

**Table 1:** The parameters extracted from the fitting of the forward bias *I*–*V* characteristics for the annealed single nanorod *n*-ZnO/*p*-GaN heterojunctions on the FIB-patterned substrate.

Annealing Temperature [°C]	*R_shunt_* [Ohm]	*I_0_*^a)^ [A]	*η* ^b)^	*k* ^c)^	*β* ^d)^	*R_ser_* [Ohm]
initial1	3 × 10^12^	4.2 × 10^−15^	18	3.2 × 10^−15^	6.5	9 × 10^7^
200	3 × 10^12^	2.5 × 10^−15^	17	1.4 × 10^−14^	6.5	7.7 × 10^7^
400	3 × 10^12^	8 × 10^−18^	10	3 × 10^−13^	6	5.5 × 10^7^
600	3 × 10^12^	1.1 × 10^−24^	4.5	5.9 × 10^−12^	5	5.5 × 10^7^

^a)^ Reverse bias saturation current; ^b)^ ideality factor; ^c)^ pre-exponential factor of space-charge-limited current law; ^d)^ exponent of the space-charge-limited current law.
